# The Aesthetic Legacy of Evolution: The History of the Arts as a Window Into Human Nature

**DOI:** 10.3389/fpsyg.2021.787238

**Published:** 2021-11-24

**Authors:** Aaron Kozbelt

**Affiliations:** Department of Psychology, Brooklyn College of The City University of New York, Brooklyn, NY, United States

**Keywords:** evolution, aesthetics, art history, creativity, modernism, postmodernism

## Introduction

Across millennia and societies, the aesthetic diversity of human imaginative culture appears stunning. This tendency is especially pronounced today, where the arts seemingly manifest unprecedented variety. It is appealing to regard human artistry as literally unbounded in its creative potential.

In this opinion piece, I argue that this unbounded view of human aesthetics is illusory. The contingent evolutionary basis of our humanity implies that our aesthetic and creative capacities are biologically constrained in fundamental ways (for a variety of views, see Graham, 2013; Kozbelt, 2017a, 2019a, 2020, in press; see also Pinker, 1997, 2002; Wilson, 1998; Dissanayake, 2007, 2015). The apparent multiplicity of the arts can be largely understood by, if not fully reduced to, a suite of evolved mental mechanisms and “aesthetic primitives” (Dissanayake, 2015), which guide the production and reception of art in ways congruent with human biology. These include peak shift (a stronger response to exaggerated stimuli), metaphor, habituation (a diminished preference for unsurprising stimuli), and semantic association (e.g., Ramachandran and Hirstein, 1999; Martindale, 2007). Such mechanisms plausibly undergird numerous statistical regularities evident in common features of aesthetic products. These include a center balancing point (Firstov et al., 2007) and scale-invariant distribution of spatial frequencies (Graham and Redies, 2010) in visual art, meter and rhyme in poetry (Keyser, 2020), and common pitch intervals, tonal hierarchies, principles of grouping and meter, and aspects of melodic contour in music (Trehub, 2000; Savage et al., 2015; Purves, 2017). Moreover, many empirical studies have found substantial cross-cultural concordances in aesthetic preference and judgment, again implying broad commonalities rather than silos of aesthetic activities so divergent that they are mutually incomprehensible (Ford et al., 1966; Chen et al., 2002; Dutton, 2009).

These lines of evidence are mainstays of evolutionarily informed views of the arts. Their central claim is that the mentalities of aesthetic creators and audiences are highly structured by the legacy of evolution, whether through natural selection (Orians, 2014), sexual selection (Miller, 2000), or as a byproduct of genuine adaptations (Pinker, 1997)—the distinction among these not being crucial to the basic point. Artistic styles are not completely determined by external cultural forces but rather follow evolved biological constraints. For instance, it is easier to mentally process mathematically simple (vs. complex) pitch intervals, and musical tonality systems based on simple intervals pervade many cultures (Purves, 2017). Importantly, evolutionary perspectives are not fully reductive about the arts and do not rule out some impact of culture (Dissanayake, 2015). Moreover, biological or evolutionary perspectives need not entail a static, one-size-fits-all ideal aesthetic target, nor preclude some long-term change (Laland et al., 2015; Nadal and Chatterjee, 2018). A capacity for aesthetic flexibility can blossom into different modes over time, even as biologically based aesthetic biases act as guardrails on that emerging variability.

In this opinion piece, I argue that documented regularities in the historical (vs. pre-historical) development of artistic styles themselves represent a neglected line of evidence that can further support and inform art's biological basis. I then discuss how biological or evolutionary views might address the challenges posed by contemporary modernism and postmodernism, two culturally prominent anti-traditional movements which undergird the contemporary ‘crisis' of the fine arts (Pinker, 2002). I close by highlighting the usefulness of identifying aesthetic regularities that have emerged—and continue to emerge—in historical times, and their implications for a biological view of the arts today.

## How Art History Informs Human Nature

The degree to which human aesthetics are constrained by our evolutionary past has strong implications for artistic traditions' trans-historical unfolding. An exclusively culture-driven, “blank-slate” aesthetic mode has few built-in constraints and should yield a history with minimal systematic long-term trends. In contrast, an evolutionary view, constrained by basic psychobiological mechanisms, implies significant structure in the development of the arts in historical times, as these mechanisms play out in predictable ways.

How aesthetic traditions unfold has long been of interest in the humanities. For instance, Elkins (2002) discussed several possibilities for visual art: a linear sequence of stylistic periods (e.g., Renaissance, Baroque, or Modernism), oscillations (e.g., alternating between Classical and Baroque modes), and life histories (as traditions establish themselves, blossom, mature, and decline). Sequences and oscillations are consistent with a socio-cultural or blank-slate view, generating variation but not directional long-term change. In contrast, life history models à* la* Elkins show clear directionality and are thus perhaps more promising targets for evolution-informed analysis. Life history trajectories are typically arch-shaped, tracing the development then decline of a tradition, but other paths—like a linear or accelerating increase over time—are possible.

Style trends can be parsimoniously understood via psychobiological processes deployed by individuals to achieve their creative and aesthetic goals (Kozbelt, 2017a). Such processes drive the most thorough scientific account of trans-historical style changes in the arts, that of Martindale (1990). In this theory, artistic creators seek critical attention for their productions. Because of habituation—a diminished preference for unsurprising stimuli—the appeal of new productions declines. Thus, creators must continually generate work that is more attention-garnering, or higher in “arousal potential.” The arousal potential of a stimulus is determined by its psychophysical intensity, capacity for meaning, and collative properties like novelty, complexity, and surprise value (Berlyne, 1971). The use of collative properties is the most effective means to attract attention, by producing more unusual combinations of ideas within an artistic style via “primordial cognition” (loosely associative, daydream-like thought), or by developing a new style altogether. Abundant evidence (detailed in Martindale, 1990) indicates that arousal potential always increases over time, as creators try to outdo their predecessors and contemporaries. Primordial cognition and stylistic change simultaneously increase overall and oscillate inversely with each other as they ascend—since only one or the other method is necessary to further increase arousal potential, and “too much” novelty is counterproductive. Martindale's (1990) extensive empirical support for his model, drawn from many artistic traditions and domains, includes laboratory experiments, ratings of artworks, and computer-based analyses of characteristics of poetic texts (see also Martindale, 1973, 1994).

Martindale's (1990) visionary theory is sadly neglected these days (Kozbelt, 2017b), but it remains without peer in its synthetic range and quantitative rigor. Resurrecting the theory in the context of big data and contemporary evolutionary aesthetics would allow a timely refocus on key questions about human nature and the arts and would complement other empirical approaches and address pressing questions about our current cultural situation.

For instance, one provocative question is how Martindale's (1990) trans-historical findings relate to Elkins's (2002) life history models. Life history models have a natural arc, out of whose ruins new traditions presumably emerge. In contrast, Martindale's variables—especially arousal potential—continually increase over time and have little directly to do a with sense of eminence or achievement, even though that constitutes the essence of a style's narrative history. Consider the most prototypical life history account, Vasari's (1550/1996) chronicle of Italian Renaissance visual art, which plays out in just those terms. It begins with Giotto's break from the long-dominant Byzantine style around the year 1300, gradually progresses, and culminates some two centuries later in the unsurpassable Michelangelo, after whom art declines. Synthesizing these two areas: How is arousal potential related to ultimate individual eminence (Murray, 2003)? How might the relations between such variables change throughout the span of a tradition? When an early pioneer like Giotto retains renown despite being superseded on some criteria (like realism), is that due to some cultural narrative about his pivotal historical importance, or does it speak to an inherent greatness in his art that survives him? What happens when a tradition declines into decadence, such that initially more attention-grabbing work may not reflect greater renown or long-term staying power? Such questions need not remain unanswered; grafting careful quantitative assessments (Martindale, 1990; Murray, 2003) onto humanistic perspectives (e.g., Elkins, 2002) provides a means to use art historical data to bolster and refine the case for a psychobiologically informed view of the arts in varied cultural contexts.

## Late Art: Modernism, Postmodernism, and Endgame(?)

Another challenging issue involves the later stages of artistic traditions. This has special relevance in today's artworld, which—despite its variety—is sometimes regarded as exhausted (Danto, 1964; Martindale, 2009). Over the last century, avant-garde artistic movements have challenged nearly every traditional aspect of the arts—precisely the aspects that seem most biologically grounded (Pinker, 1997, 2002; Wilson, 1998). Early modernist creators abandoned traditional rule systems based on natural aesthetics and invented new personal aesthetic systems (Keyser, 2020). Postmodernism is perhaps even more radical, involving a pluralistic milieu of heterogeneous styles, short-lived artistic movements, idiosyncratic and one-trick creators making desperate ploys for originality, a suspension of value judgments, and a lack of settled meaning (Elkins, 2002). Do modernist and postmodernist movements represent a final liberating triumph of blank-slate socio-cultural forces over biology? Are biologically evolved constraints on the arts still relevant?

One might approach this issue by asking if documented trends from the past—like Martindale's (1990) trajectory of increasing arousal potential—are sustainable indefinitely. Martindale's own answer was an unequivocal no, echoing the second law of thermodynamics: as styles change, disorder and unpredictability always increase. Thus, aesthetic entropy will inexorably exhaust from within any aesthetic tradition worthy of the name.

The fundamental endgame bottleneck is the inability of the fine arts in their late stages to meaningfully communicate to audiences. On this note, Martindale (2009) described art's tragic end, claiming that the high arts are already extinct. Murray (2003) likewise argued that accomplishments in the arts have been in a state of decline since at least the 19th century. Less cynically, Keyser (2020) argued that that some aesthetic rules are simply more natural for humans to process than others. This insight raises provocative questions about human nature and the contemporary arts: a research emphasis on identifying aesthetic rules, a distinction between natural and unnatural rules, and a further distinction between modernist codes that represent genuine historical discoveries of new natural aesthetic principles (like recursion in the poetry of Wallace Stevens or fractals in Jackson Pollock's drip paintings) vs. those that Keyser claims do not (like Arnold Schoenberg's musical serialism).

## Newly Emergent Aesthetic Principles

Keyser's (2020) notion of aesthetic codes provides a means to understand why some artistic trends rapidly wane, while other aesthetic principles, which have emerged in historical (rather than pre-historical) times, have endured (Kozbelt, in press). Persistent principles include poetic recursion, certain orthographic systems, compositional balancing points or linear perspective in painting, diatonic harmony and counterpoint in music, and numerous structural or formal devices for organizing aesthetic raw material across domains. Are these different fates preordained by our evolved aesthetic sense and an inherent adaptiveness of certain aesthetic modes (see Gombrich, 1979), or is a *post-hoc* socio-cultural account the best we can do?

Disentangling these threads is challenging but not impossible. If modernism or postmodernism resembles other artistic periods, then similar patterns should be evident in quantitative measures like the strongly positively skewed distribution of creative achievement (Murray, 2003) and trans-historical style trends (Martindale, 1990). Computational models of cultural evolution (e.g., Creanza et al., 2017), sophisticated assessments of contemporary conceptual art from biological perspectives (e.g., Kranjec, 2015; De Tiège et al., 2021), and documentation of the ongoing codification of practice within new artistic technologies, like photography or cinema (e.g., Edgar et al., 2015), can also inform how new aesthetic regularities arise in a contemporary cultural context. However, if modernism or postmodernism truly represent something qualitatively different, there is little reason to expect the past to predict the future, and perhaps then the relevance of biological evolution to art really has evaporated.

Another possibility is that the broad-brush picture of the arts today is not so different from the past: a complex cultural system consisting of many niches with different characteristics and dynamics and audiences and selection pressures, but numerically dominant popular forms partaking of humanistic natural aesthetic principles, with hedonic enjoyment of beauty and meaning-making paramount in aesthetic experience (Kozbelt, in press). If the contemporary high arts are irrelevant for most people, this reinforces the enduring power of long-standing evolutionary aesthetic principles and suggests we might refocus research onto popular art forms (Pinker, 1997, 2002).

Empirically addressing such issues would constitute a productive, provocative research agenda. Testing the assertion that our biological inheritance remains relevant to the contemporary arts (see Kozbelt, 2019b, 2020, in press), even if we cannot yet discern exactly how, has the potential to further illuminate the evolutionary basis of our aesthetics and human nature itself.

## Author Contributions

The author confirms being the sole contributor of this work and has approved it for publication.

## Conflict of Interest

The author declares that the research was conducted in the absence of any commercial or financial relationships that could be construed as a potential conflict of interest.

## Publisher's Note

All claims expressed in this article are solely those of the authors and do not necessarily represent those of their affiliated organizations, or those of the publisher, the editors and the reviewers. Any product that may be evaluated in this article, or claim that may be made by its manufacturer, is not guaranteed or endorsed by the publisher.
